# High-Fat, High-Calorie Breast Milk in Women with Overweight or Obesity and Its Association with Maternal Serum Insulin Concentration and Triglycerides Levels

**DOI:** 10.3390/children11020141

**Published:** 2024-01-23

**Authors:** Michael G. Ross, Manasa P. Kavasery, MacKenzie K. Cervantes, Guang Han, Bernardo Horta, Kelly P. Coca, Suleyma O. Costa, Mina Desai

**Affiliations:** 1 Department of Obstetrics and Gynecology, David Geffen School of Medicine, University of California Los Angeles at Harbor-UCLA, Torrance, CA 90502, USA; mdesai@lundquist.org; 2 The Lundquist Institute at Harbor-UCLA Medical Center, 1124 West Carson Street, RB3 Building, Torrance, CA 90502, USA; mkavasery@dhs.lacounty.gov (M.P.K.); mkerr@lundquist.org (M.K.C.); ghan@lundquist.org (G.H.); 3 Department of Obstetrics and Gynecology, Charles R. Drew University, Los Angeles, CA 90059, USA; 4 School of Medicine, Universidade Federal de Pelotas, Pelotas 96010-610, Brazil; blhorta@ufpel.edu.br; 5 Escola Paulista de Enfermagem, Universidade Federal de São Paulo, São Paulo 04023-900, Brazil; kcoca@unifesp.br; 6 Laboratory of Metabolic Disorders, School of Applied Sciences, University of Campinas, Campinas 13083-970, Brazil; s179250@dac.unicamp.br

**Keywords:** milk intake, triglycerides, insulin, fatty acids, foremilk and hindmilk breast milk

## Abstract

The childhood obesity epidemic continues to be a challenge. Maternal obesity and excessive infant weight gain are strong predictors of childhood obesity, which itself is a major risk factor for adult obesity. The primary source of nutrition during early life is breast milk, and its composition is impacted by maternal habitus and diet. We thus studied the relationship between maternal BMI, serum lipids and insulin, and breast milk fat and calorie content from foremilk to hindmilk. Women who were exclusively breastfeeding at 7–8 weeks postpartum were BMI classified as Normal (18.5–24.9, *n* = 9) and women with Overweight/Obese (OW/OB ≥ 25, *n* = 13). Maternal blood and continuous breast milk samples obtained from foremilk to hindmilk were analyzed, and infant milk intake was assessed. Women with OW/OB had significantly higher milk fat and calorie content in the first foremilk and last hindmilk sample as compared to Normal BMI women. Amongst all women, maternal serum triglycerides, insulin, and HOMA were significantly correlated with foremilk triglyceride concentration, suggesting that maternal serum triglyceride and insulin action contribute to human milk fat content. As the milk fat content of OW/OB women has caloric implications for infant growth and childhood obesity, these results suggest the potential for modulating milk fat content by a reduction in maternal serum lipids or insulin.

## 1. Introduction

Rates of adult overweight and obesity (OW/OB) have dramatically increased during the past 40 years, resulting in a true obesity epidemic. Currently, approximately 73% of US adults are overweight or obese and 42% are obese [1]. Obesity now accounts for more deaths than cigarette smoking [2]. The epidemic of adult obesity has extended to childhood and adolescence. Childhood overweight and obesity prevalence rates are high across all racial groups, with particularly increased rates among Hispanic children [3,4,5,6]. Hispanic children are likely to exhibit an earlier onset of obesity and be severely obese [7] as compared to their non-Hispanic, White peers. From 2003–2004 to 2015–2016, the prevalence of obesity increased from 16.8% to 18.5% among U.S. youth (2–19-year-old), while it increased strikingly to 25.8% among Hispanic youth [8]. In addition to factors contributing to childhood OW/OB, the recent COVID epidemic has correlated with a further rise in childhood obesity [9].

Of greater concern, is that numerous reports have confirmed that infant obesity predicts childhood obesity, which itself is a major risk factor for adult obesity [10,11,12,13,14,15,16,17,18]. Numerous studies demonstrated a link between prenatal exposure to maternal obesity or excessive gestational weight gain and early childhood obesity [19,20,21,22,23]. Specifically, maternal OW and OB result in the odds ratio of overweight 2-year-old children (BMI for age-sex percentile > 85%) of 1.50 and 2.34, respectively [24]. 

In part, maternal obesity increases the risk of childhood and adult obesity as a result of altered in utero hypothalamic development resulting in increased appetite and reduced satiety neurons, and thus, early life hyperphagia [25,26]. Compounding this process of developmental programming, early life nutrition resulting in excessive infant weight gain further contributes to offspring obesity. Notably, being at the highest quintile of weight gain during early infancy (birth to 5 months of age) increased the risk of being overweight by 2-fold at 4.5 years [13] and rapid infant weight gain (>1 SD) was associated with a 5-fold risk of obesity at age 20 [15]. 

Thus, infant nutrition, whether by breast milk or formula, has important consequences for later life health. The American Academy of Pediatrics recognizes breastfeeding and human milk as the “normative standards for infant feeding” and recommends exclusive breastfeeding for 6 months, in accord with the 2011 Surgeon General’s Call to Action to Support Breastfeeding, the CDC, and the WHO. Nearly 80% of women (2011–2015) begin breastfeeding at birth and over a quarter of infants receive some breastfeeding until 12 months of age [27,28,29]. Thus, most infants are exposed to breast milk sometime during the first 6 months of life.

The benefits of human milk versus formula are well recognized [30,31]. Breastfed infants have a lower incidence of infectious diseases, such as diarrhea [9], otitis media [32], and lower respiratory tract illness [33] compared with formula-fed infants. Further, feeding higher volumes of formula in early infancy is associated with a greater body weight gain and being overweight in later infancy [34,35]. There have been few rigorous studies of human milk composition or energy content in OW/OB women [36], of quantified newborn intake of human milk from normal BMI or OW/OB women [37], or the effect of total caloric intake on infant weight gain in any group. Whereas most studies of breast milk have examined single, timed samples of expressed milk, we sought to examine the entire content of the breast, from foremilk to hindmilk, in Normal BMI and OW/OB women, while controlling for time of day and weeks postpartum. Our results show that the breast milk of OW/OB women has a significantly higher fat and calorie content. Based on the reported literature, it can be predicted that a higher energy supply in the early period of life may promote early fat accrual and obesity later in life [36]. The association of milk calories and fat content with maternal serum lipids and select hormones suggest opportunities for modulating milk composition for infants at risk of OW/OB via maternal dietary intervention [38,39] or pharmacologic approach [40]. 

## 2. Materials and Methods

*Study Participants*: This study was approved by the Institutional Review Board at the Lundquist Institute, at Harbor-UCLA Medical Center. We enrolled women delivering singleton term pregnancies after written human subjects’ consent was achieved (Study protocol #32035-01, Approval date 22 April 2021). Exclusion criteria include women with breast implants, prior breast surgery, flat/inverted nipples, tongue-tie or low birth weight infants (<2500 g), or pregestational diabetes. Women were selected for BMI (based on pre-pregnancy reported weight) 18–24.9 (Normal BMI) and ≥25 (overweight/obese). All mothers were committed to exclusive breastfeeding for at least two months. Studies were performed at 7–9 weeks postpartum (mature milk). Sample size (80% power, alpha 0.05), based upon our preliminary data of predicted mean fat content of 1.5 ± 0.2 and 1.8 ± 0.2 g/dL in Normal BMI and OW/OB groups, respectively, required seven patients in each group. 

Women were consented for enrollment and interviewed by staff for demographic information, including maternal age, ethnicity, and pre-pregnancy height and weight. Additional demographic data were obtained from the electronic medical record or patient interview, including height and weight at the last prenatal visit, gestational age at delivery, pregnancy complications (including gestational diabetes), medications, mode of delivery, and baby’s length and weight at birth and discharge and gender. 

All studies were performed in the outpatient clinic of the Lundquist/Harbor-UCLA Clinical and Translational Research Center (CTRC) between 10 a.m. and 12 p.m. to avoid potential circadian changes in breast milk composition. Mothers were fasted for ≥1 h prior to study. Prior to initiation of nursing, maternal blood samples were drawn for analysis.

Breast milk samples were obtained at least ≥1.5 h from the prior infant breastfeed, which was from only one breast. The opposite breast was used for breast milk sampling. The nipple and areola were wiped clean and an electrical pump (Medela, McHenry, IL, USA) was applied to the breast. Breast milk samples were obtained continuously in 10 mL aliquots, and pumping continued until primary breast was emptied or there was no further milk production. Milk samples were analyzed by Miris (Uppsala, Sweden) milk analyzer and remaining samples were frozen for further analysis. 

*Infant Anthropometric Measurements*: The infant’s body weight and length were measured using a Seca 757 electronic baby scale and SECA 416 infantometer (Seca Ltd., Hamburg, Germany), respectively. Infants wearing a fresh dry diaper of known weight (20 g pampers, size 1) were weighed to the nearest 1 g. Three measurements of supine length to the nearest 0.1 cm were obtained and the average was used to reflect the length. Infant ponderal index (PI) was calculated by dividing weight (kg) by length cubed kg/m^3^, a more accurate adiposity parameter during infancy [41]. 

*Milk Intake*: Following the pumping, the infant was then allowed to feed (from the breast not used for milk sampling) and then reweighed to determine milk intake during a single feed. 

*Blood Analysis*: Serum samples were sent to Quest Diagnostics for analysis of the lipid panel (triglycerides, total cholesterol, LDL-Cholesterol, HDL-Cholesterol, non-HDL Cholesterol, Cholesterol/HDL Ratio), glucose and insulin. Invitrogen (Waltham, MA, USA) ELISA kits were used to analyze plasma leptin (KAC2281; sensitivity < 0.003 ng/mL, inter-assay and intra-assay coefficient variability 4.6% and 3.6%, respectively) and adiponectin concentrations (KHP0041; sensitivity < 0.0001 µg/mL, inter-assay and intra-assay coefficient variability 5.8% and 3.5%, respectively). 

*Milk Analysis*: Fresh milk samples were analyzed for fat, protein, and carbohydrate content using the ‘Miris Human Milk Analyzer’ (Miris AB, Uppsala, Sweden). All samples were kept at 40 °C (Miris Heater), homogenized for 1.5 s/1 mL (Miris Ultrasonic Processor) and samples were injected into the flow cell and measured in triplicate. The analyzer, which has been validated for accuracy [42], is a semi-solid, mid-infrared (MIR) transmission spectroscopy that uses specific wavebands for the determination of functional carbonyl groups (5.7 µm), amide groups (6.5 µm), and hydroxyl groups (9.6 µm) for fat, protein, and carbohydrate, respectively. True protein is adjusted for non-protein nitrogen (crude protein multiplied by 0.8), and total carbohydrate content includes lactose and oligosaccharides. Total solids are measured by the drying oven, and the analyzer provides a calculation of energy (kcal per 100 mL) using conversion factors of 4.0 (carbohydrate), 9.25 (fat), and 4.4 (protein).

*Lipid Analysis*: Milk and maternal serum lipid studies were performed at UCLA Lipidomics Core. Plasma (25 µL) and milk (25 µL) samples were centrifuged and extracted using a modified method described by Bligh and Dyer [43]. Before biphasic extraction, an internal standard mixture consisting of 70 lipid standards across 17 subclasses was added to each sample (AB Sciex, Redwood City, CA, USA, 5040156, Avanti (Alabaster, AL, USA) 330827, Avanti 330830, Avanti 330828, Avanti 791642). After two extractions, pooled organic layers were dried (Thermo, Waltham, MA, USA, SpeedVac SPD300DDA; ramp setting 4) at 35 °C for 45 min with 90 min of total run time. Resuspended lipid samples (1:1 methanol/dichloromethane with 10 mM ammonium acetate) were transferred to robovials (Thermo 10800107) for analysis. 

Samples were analyzed using Sciex 5500 with a DMS device (Lipidyzer Platform, SCIEX, Framingham, MA, USA) and an expanded targeted acquisition list comprising 1450 lipid species across 17 subclasses. Differential Mobility Device on Lipidyzer was tuned with EquiSPLASH LIPIDOMIX (Avanti 330731, Alabaster, AL, USA). Data analysis was performed using in-house data analysis platform, similar to the Lipidyzer Workflow Manager (UCLA Lipidomics Core, Los Angeles, CA, USA) [44]. Detailed instrument methods, including settings, tuning protocol, and multiple reaction monitoring list, are as previously described [44]. Quantitative plasma and milk values were normalized to volume (mLs), and concentrations are reported as nmoles/mL.

*Statistical Analysis*: An unpaired t-test was used for data analysis. Milk composition (first, middle, last) was analyzed by repeated measures of ANOVA. Linear regression was used to test if maternal serum triglycerides (TG) and insulin concentrations significantly correlated with milk TG content. The Shapiro–Wilk test was used to check for normality. Values are expressed as means ± SEM. All analyses included *n* = 9 Normal BMI and *n*= 13 OW/OB mother–infant, except for lipidomics, which included *n* = 9 Normal BMI and *n* = 11.

## 3. Results

### 3.1. Maternal Characteristics

A total of 22 women were enrolled in the study, of which 9 were Normal (BMI 21.2 ± 0.6) and 13 were OW/OB (BMI 32.5 ± 1.5). Subjects’ demographics included Asian (5), Hispanic (12), Black (4) and White, non-Hispanic (1). The infant gender was 10 male and 12 female. Maternal fasting (1 h) serum values are depicted in Table 1. Among the serum values, insulin and leptin were significantly increased and HOMA-IR (2.87 ± 0.70 vs. 1.87 ± 0.54, *p* < 0.08) showed an increased trend in OW/OB subjects. Notably, serum TG, cholesterol and adiponectin concentrations were similar in Normal BMI and OW/OB subjects.

### 3.2. Milk Composition

**Figure 1:**
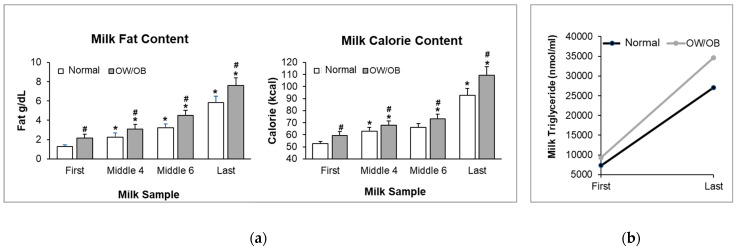
Relationship between maternal BMI and milk fat and calorie content: (**a**) fat and calorie content of first, middle and last milk samples from Normal BMI and OW/OB women. Values are mean ± SEM of *n* = 9 (Normal BMI) and *n* = 13 (OW/OB) groups. ^#^ *p* < 0.05 OW/OB vs. Normal BMI; * *p* < 0.05 vs. first milk sample. (**b**) The first milk sample’s TG content predicts the last milk sample’s TG content.

The total pumped milk volume (i.e., breast volume) was similar in the two groups. Milk composition was examined in the first, the fourth, sixth and last 10 mL samples from the pumped breast. As demonstrated in Table 2, crude and total protein, and carbohydrate and solids concentrations were similar between Normal BMI and OW/OB women. However, milk fat and caloric content were significantly increased in OW/OB women (Repeated ANOVA Fat: F(1, 2) = 7.78, *p* < 0.01; Calorie: F(1, 2) = 8.39, *p* < 0.005). Post hoc analysis demonstrated significant differences between the BMI groups as well as between the first and last samples (Figure 1a). Notably, when averaged over the four samples, caloric content values were 13% greater in OW/OB women.

In both Normal BMI and OW/OB subjects, from the first to last sample, milk fat concentration increased 3- to 4-fold (Figure 1b), with mid samples representing intermediate values. Caloric content similarly increased nearly 2-fold from the first to the last sample (Figure 1a). Crude and true protein and carbohydrate and solid content remained unchanged from the first to last sample (Table 2).

### 3.3. Lipid Analysis

**Figure 2:**
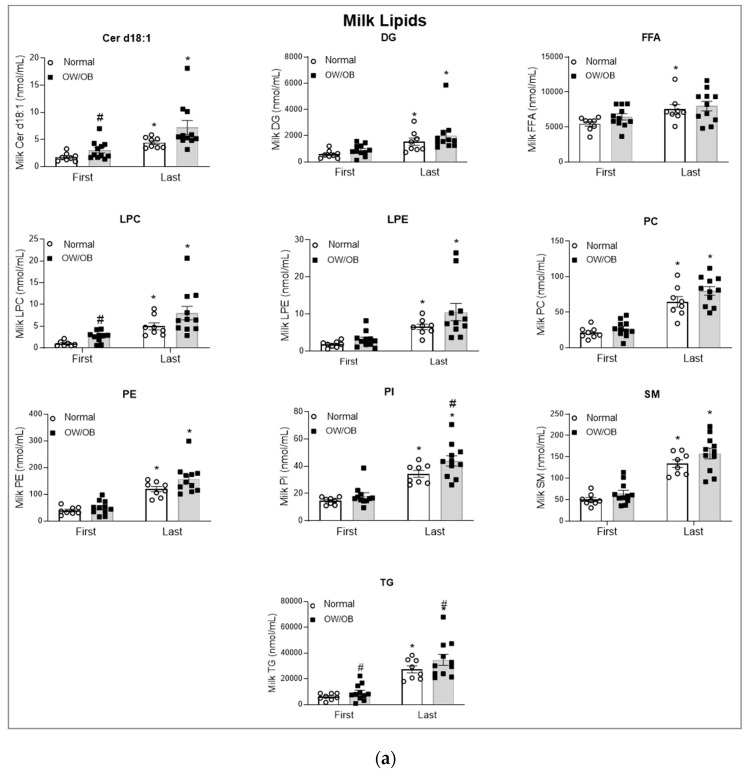

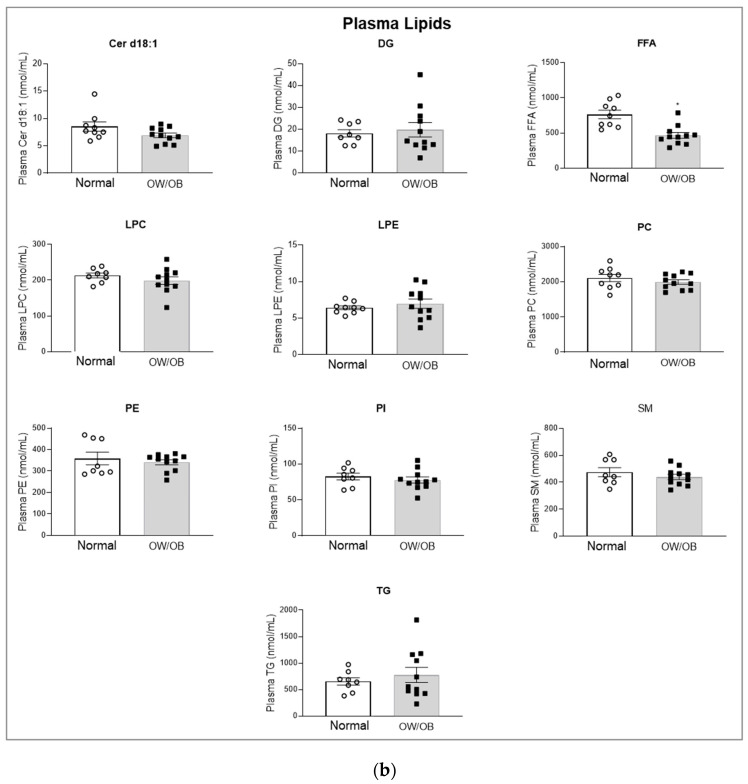
Milk and maternal plasma lipids: (**a**) Lipid profile of first and last milk samples and (**b**) plasma from Normal BMI and OW/OB women. Values are mean ± SEM of *n* = 9 (Normal BMI) and *n* = 11 (OW/OB) groups. ^#^ *p* < 0.05 OW/OB vs. Normal BMI; * *p* < 0.05 last vs. first milk sample. Abbreviations: ceramide (Cer d18:1), diacylglycerols (DG), free fatty acids (FFA), lysophosphatidylcholine (LPC), lysophosphatidylethanolamine (LPE), phosphatidylcholine (PC), phosphatidylethanolamine (PE), phosphatidylinositol (PI), sphingomyelin (SM) and triglycerides (TG).

As compared to Normal BMI subjects, OW/OB subjects had significantly increased milk TG in both the first and last sample (Figure 2a).

**Figure 3:**
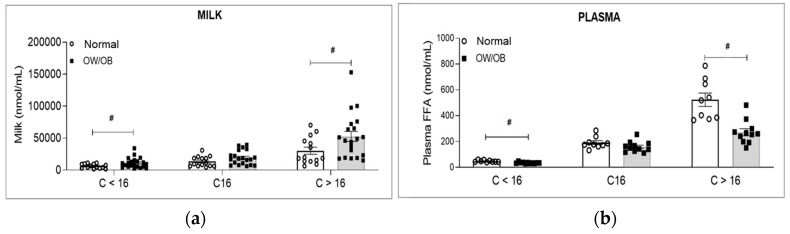
Milk and maternal plasma fatty acids by chain length: (**a**) fatty acid concentration of combined first and last milk samples and (**b**) plasma from Normal BMI and OW/OB Women. Values are mean ± SEM of *n* = 9 (Normal BMI) and *n* = 11 (OW/OB) groups. ^#^ *p* < 0.05 OW/OB vs. Normal BMI. Medium-chain FAs (C < 16), palmitic acid (C16) and long-chain FAs (>16).

In assessing the increase in milk fat concentration lipids from the first to last sample, milk lipid analysis demonstrated significantly increased milk TG. As compared to first milk sample, the last sample demonstrated increased concentrations of free fatty acids (FFA), neutral lipid (diacylglycerols, DG), ceramide (Cer d18:1), and phospholipids (phosphatidylcholine, PC; phosphatidylethanolamine, PE; phosphatidylinositol, PI; lysophosphatidylcholine, LPC; lysophosphatidylethanolamine, LPE; Sphingomyelin, SM). In all cases, OW/OB showed an increased trend though only Cer d18:1, and LPC were significantly higher in OW/OB first milk as compared to Normal BMI subjects (Figure 2a). FFA analysis by chain length demonstrated that medium- (C < 16) and long-chain FAs (>16) were significantly higher with a trend towards increased levels of C16 (palmitic acid) in milk from OW/OB as compared to Normal BMI women (Figure 3a).

In contrast to milk lipids, maternal plasma lipid analysis showed no differences except for total FFA (Figure 2b) and serum medium- and long-chain FAs, (Figure 3b) which were significantly decreased in OW/OB versus Normal BMI subjects.

### 3.4. Maternal Serum Factors and Milk Triglycerides (TG)

**Figure 4:**
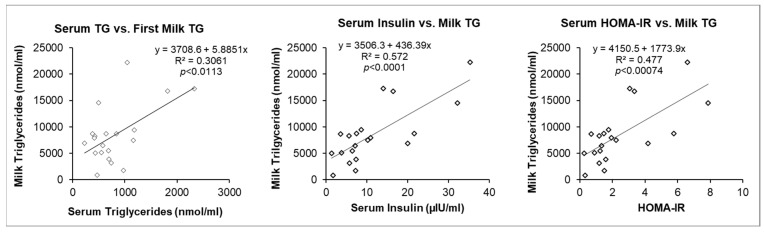
Linear regression of maternal Serum vs. first milk sample: serum TG vs. milk TG and serum insulin and HOMA-IR vs. milk TG. *n* = 20 of combined OW/OB and Normal BMI groups. Triglycerides (TG).

A linear regression analysis of select maternal factors versus milk TG was performed to assess possible mechanisms contributing to high fat content (Figure 4). Maternal insulin concentration (R^2^ = 0.572, F(1, 18), *p* < 0.0001) and HOMA-IR (R^2^ = 0.477, F(1, 18), *p* < 0.0007) were highly correlated with first milk TG (Figure 4), while maternal serum TG was moderately correlated (R^2^ = 0.306, F(1, 18), *p* < 0.01).

### 3.5. Low versus High Milk Fat Content

**Figure 5:**
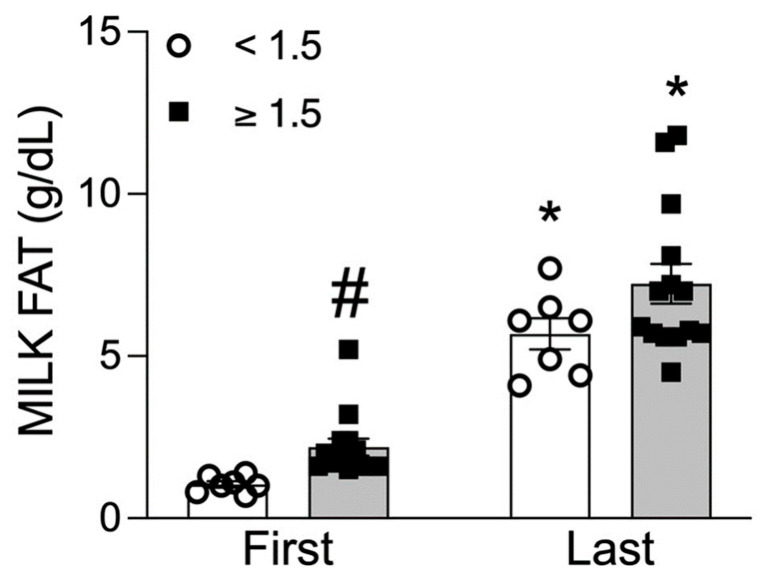
Low and high milk fat content: values are mean ± SEM of *n* = 7 (Low) and *n* = 15 (High) milk groups with approximately 80% of OW/OB and 20% of Normal BMI women being in high milk fat group. * *p* < 0.05 first vs. last; ^#^ *p* < 0.05 low vs. high milk fat.

The subjects were further divided into two groups based upon the fat content of the first milk sample (Low-Fat, High-Fat). The Low-Fat group (*n* = 7) which started at <1.5 g/dL, ended with lower milk fat and TG whereas the High-Fat group (*n* = 15) that started at ≥1.5 g/dL ended with higher milk fat and TG (Figure 5). Amongst the OW/OB women, ~80% and amongst Normal BMI, ~20% were identified as having High-Fat content based upon foremilk assessment.

### 3.6. Infant Characteristics

Combined male and female data are depicted in Table 3. Infants of OW/OB mothers demonstrated a trend toward increased birth weight and increased ponderal index, with male infants of OW/OB mothers having a greater ponderal index than males of Normal BMI mothers (2.85 ± 0.05 vs. 2.42 ± 0.08, *p* < 0.05). As described above, following the pumping of the study breast, infant feeding studies were performed on the alternate breast. Despite similar body weight at study (OW/OB: 5125 ± 212; Normal BMI: 5290 ± 185), the milk intake of infants of OW/OB mothers was markedly greater than that of Normal BMI mothers (90.0 ± 13.6 vs. 64.5 ± 6.4 g, *p* < 0.05), though there was no difference between males and females within each group. Notably, the frequency of feedings was comparable in infants of Normal BMI women and women with OW/OB (day: every 2.2 ± 0.2 vs. 2.0 ± 0.2 h; night: every 4.0 ± 0.2 vs. 4.1 ± 0.3).

## 4. Discussion

The present results are unique in demonstrating that women with OW/OB have increased milk fat and calories, spanning foremilk to hindmilk, as compared to Normal BMI women. The main findings of the study demonstrate that; (1) women with OW/OB have increased TG, fat, and calories, with markedly increased milk medium-chain FAs (<C16) and long-chain FAs (>C16); (2) maternal foremilk TG is highly correlated with hindmilk TG levels; (3) and amongst all women (irrespective of maternal BMI), maternal serum insulin and HOMA are correlated highly, and maternal serum TG modestly correlated with foremilk TG concentration. Together, these findings suggest that maternal serum insulin, and likely TG action, contribute to human milk fat content.

On average, human milk contains 0.8–0.9% protein, 3–5% fat, 6.9–7.2% carbohydrates, and 0.2% minerals. Median total caloric content is 66 kcal/100 mL [45,46,47,48], with an interquartile range of 62.0 to 72.5 kcal/100 mL, indicative of individual variance. Although there are few studies, previous results suggest that maternal BMI is correlated with milk caloric content [47,48,49]; though, most of the prior studies examined a single timed or random breast milk sample. Other studies have demonstrated that milk fat increases from foremilk to hindmilk [50,51,52]. As recently emphasized in a systematic review, standardized protocols for the collection and analysis of human milk samples are essential to ensure representative samples that can be compared across studies [53]. 

Familial lifestyle and environmental factors, including developmental programming effects of the maternal environment during both in utero [25,54] and newborn periods (i.e., lactational programming), result in a predisposition to offspring obesity in women with OW/OB and may contribute to the obesity epidemic [55,56,57]. In the present study, breast milk of OW/OB women demonstrated significantly increased fat and caloric content. Serum FFAs were significantly reduced among OW/OB subjects, though there were no other differences in serum lipids between Normal BMI and OW/OB subjects. Due to maternal lifestyle limitations with infant care and early morning infant feeds, we did not collect blood samples after overnight fasting. Most, but not all [58] studies have demonstrated increased serum TG and cholesterol in OW/OB nonpregnant [59,60] and postpartum [61] women, though lactation appears to reduce serum lipid levels. The lack of difference in serum lipids potentially may reflect the effects of morning food intake prior to the study [62].

Despite the absence of serum FFA and lipid differences, OW/OB women demonstrated significantly increased fat content from the first to last breastmilk sample. TGs make up 98% of milk lipid content and contribute 40–50% of human milk calories [63]. Human milk TGs result from three sources: endogenous fat stores, dietary lipids, and de novo mammary epithelial cell (MEC) synthesis. Endogenous fat stores or dietary lipids modulate serum lipids and, thus, contribute to milk long-chain FAs, as human MECs have limited ability to synthesize C18 FAs. These long-chain FA, which contribute ~80% of the total milk fat content, primarily are carried by very low-density lipoproteins and are taken up by MECs following hydrolysis by MEC lipoprotein lipase. MEC de novo synthesis contributes short/medium-chain FAs (C6–C16) with acetate as the primary carbon source. Whereas a maternal high-fat diet may increase total milk fat as a result of long-chain FAs [64], a low-fat diet increases MEC synthesis of short- and medium-chain FAs. The present results demonstrating both increased medium- and long-chain FA’s in the milk of women with OW/OB indicates that the high-fat content of milk is likely a result of both de novo mammary epithelial cell (MEC) and uptake of preformed FAs [65,66]. 

Studies show that increased maternal dietary fat intake increases breast milk fat content fat [67,68] with no impact on milk lactose or protein [69]. Limited studies also suggest that maternal dietary/supplement intake of some micronutrients, including fat-soluble vitamins (B1, B2, C) were reflected in the breast milk composition [70,71]. Thus, maternal diet impacts milk composition, though with a delay of ~24–36 h [70,72]. Although it is possible that maternal serum TG differences existed throughout the day prior to the milk sampling, the present results suggest that factors in addition to BMI may regulate milk fat composition. Linear regression analysis supports the strong association of the first milk sample with serum insulin concentrations and serum TG levels. Thus, insulin resistance, consistent with the association of first milk TG with HOMA-IR values, may be etiologic in first milk TG content. 

Evidence suggests that insulin resistance occurs unequally among primary insulin-target sites, while the mammary breast is extremely sensitive to insulin during lactation [73]. We propose the hypothesis that OW/OB patients with insulin resistance have proportionately less insulin resistance at the lactating breast as compared to liver, adipose, and muscle. Thus, the relatively increased serum insulin concentrations in these patients result in increased local mammary epithelial cell effects, promoting both de novo lipogenesis and cellular fatty acid uptake and incorporation into milk. This is consistent with the known role of insulin in stimulating the enzymes of de novo lipogenesis (fatty acid synthase) and FA uptake, including mammary gland lipoprotein lipase [74,75]. Despite the strong correlation with foremilk TG, neither serum insulin nor serum TG concentrations were significantly predictive of last milk TG concentrations, although OW/OB subjects’ milk TG concentrations were markedly greater than Normal BMI women. Together, these findings indicate that the regulatory mechanisms of foremilk and hindmilk composition differ. 

Maternal leptin serum levels increase throughout pregnancy, and maternal serum leptin levels are higher in pregnant women with OW/OB than those with normal weight. Leptin is also produced by the placenta and relatively small amounts are secreted into the fetal circulation. The increased serum leptin levels in macrosomic newborns may be a consequence of both increased fat mass and potentially maternal or placental contributions. Thus, cord blood leptin is positively associated with maternal OW/OB [76]. It is likely that leptin resistance may develop before birth and contribute to newborn, and potentially longer-term, appetite/satiety programming.

Whether the markedly increased leptin concentrations in women with OW/OB potentially impacts MEC milk fat production is currently unknown. This premise is supported by limited studies on lactating bovine MEC that report leptin-mediated fat synthesis in the presence of prolactin [77]. Nonetheless, the precise role of maternal circulating leptin on MEC FA uptake and lipid synthesis is not clear, as studies show that leptin is also expressed and produced by human MEC [78,79]. Alternatively, other metabolic alterations in OW/OB mothers may influence milk fat production. 

Studies show that ethnicity does not influence the breast milk macronutrient (protein, fat, carbohydrate, and moisture) content [80]. However, the specific fatty acid composition has been shown to differ among ethnic groups in the same region [81]. 

The concept relating to the volume of milk is not well understood. Women with OW/OB had a slightly, but not significantly, increased volume of breast milk obtained by pumping. There have been numerous reports of challenges of breastfeeding among OW/OB women, a result of body habitus and breast size as well as infant positioning and latching [82]. Although only a single sample, these results suggest that reduced milk volume is not perceived as a difficulty in breastfeeding women with OW/OB in the current study.

In this small cohort, infant birth weight demonstrated an increased trend in OW/OB subjects, consistent with data from large cohorts [83]. Although we examined only a single feeding episode, our results indicated a nearly 40% greater milk intake among both male and female infants of OW/OB as compared to Normal BMI mothers. Infant weighing before and after feeding has an estimated accuracy of ~10% for the determination of milk intake [84]. Whether the single increased milk intake can be extrapolated to daily milk intake can only be determined with more extensive study. However, these results are consistent with animal studies from our laboratory and others [26,85,86], demonstrating that overfeeding during early developmental periods alters appetite regulation including satiety responsiveness, resulting in programmed hyperphagia among rodent offspring from OW/OB dams. 

In the current study, protein content was similar in both groups of women. A comprehensive review [87] has shown that not all studies demonstrate that higher protein intake is associated with excess body weight. Overall, four out of seven studies showed no significant impact of total protein intake on infant anthropometry. A total of three studies showed a positive impact on infant anthropometry, free fat mass, and adiposity though one of these studies also reported reversal on infant adiposity (skinfold gain) from 3 months of age onwards.

When subjects were divided into those with Low- or High-fat first milk samples, the results suggested that the amount of fat in the first milk predicts the fat content in the last milk. Thus, infants of OW/OB mothers may ingest foremilk with a higher fat/calorie content (13% increase), and a greater total volume of milk, resulting in a greater proportion of the highest fat hindmilk. These findings may contribute to increased weight gain observed in infants of OW/OB mothers [83]. Notably, the child weight-for-age Z score was significantly higher among women with increased breast milk lipid content [88]. Together, these results suggest that “responsive parenting” guidelines may be of value in preventing excessive infant weight gain [89].

Several limitations of the current study must be considered when interpreting these results. The limitations include a small sample size and a 1 h maternal fasting blood sample. Furthermore, an assessment of maternal dietary intake would provide a better overall understanding of the influence of maternal OW/OB, as well as diet, on milk composition. Lastly, in addition to maternal pre-pregnancy BMI, data on maternal weight gain during and after pregnancy would assist in evaluating their impact on milk composition.

## 5. Conclusions

Childhood and adult obesity rates continue to increase despite intensive focus and interventions at societal and individual levels. The influence of high fat/calorie content of human milk from women with overweight/obesity in contributing to excessive infant weight gain in infants with heightened orexigenic drive is less well-recognized. Breast milk has been highly promoted as ‘breast is best’, and clearly is advantageous in comparison to infant formula. Our study confirms that women with overweight and obesity produce milk with higher fat, triglyceride, and caloric content, with milk fat content increasing progressively from foremilk to hindmilk. This is consistent with markedly increased milk medium-chain FAs (<C16) and long-chain FAs (>C16). Furthermore, maternal serum insulin and triglyceride concentrations are correlated with foremilk milk triglycerides. Prevention of early infant excessive growth and the risk for generational obesity requires novel strategies for infant feeding and/or the development of “personalized breast milk”.

## Figures and Tables

**Table 1:** Maternal Characteristics.

	Normal (BMI 18.9–24.9) *n* = 9	OW/OB (BMI ≥ 25) *n* = 13	*p*-Value OW/OB vs. Normal
Age	29.8 ± 2.6	29 ± 1.9	NS
BMI	21.2 ± 0.6	31.9 ± 1.6 *	*p* < 0.001
Blood Glucose (mg/dL)	85.0 ± 3.3	84.4 ± 2.5	NS
Serum Insulin (µIU/mL)	8.4 ± 2.0	17.6 ± 3.4	*p* < 0.04
* HOMA IR	1.87 ± 0.54	2.87 ± 0.70	*p* = 0.083
Serum Triglycerides (mg/dL)	98.6 ± 20.2	101.5 ± 18.9	NS
Serum Total Cholesterol (mg/dL)	205.3 ± 14.1	185.6 ± 10.0	NS
Serum LDL-Cholesterol (mg/dL)	124.4 ± 11.3	108.8 ± 8.6	NS
Serum HDL-Cholesterol (mg/dL)	61.2 ± 5.9	56.9 ± 3.3	NS
Serum Non-HDL Cholesterol (mg/dL)	144.1 ± 11.9	128.7 ± 9.5	NS
Cholesterol/HDL Ratio	3.4 ± 0.2	3.3 ± 0.2	NS
Serum Leptin (ng/mL)	11.4 ± 2.5	32.7 ± 8.9 *	*p* < 0.001
Serum Adiponectin (µg/mL)	2.1 ± 0.2	2.6 ± 0.2	NS
Total Milk Volume (mL)	80 ± 12	88 ± 7	NS

Values are mean ± SEM. * HOMA IR (Homeostatic Model Assessment of Insulin Resistance) = insulin (μIU/mL) × glucose (mmol/L)/22.5. NS = not significant.

**Table 2:** Milk Composition.

Milk	First Sample (1st Sample)		Middle (4th Sample)		Middle (6th Sample)		Last (8th Sample)	
	Normal	OW/OB	Normal	OW/OB	Normal	OW/OB	Normal	OW/OB
Crude Protein (g/dL)	1.17 ± 0.04	1.16 ± 0.04	1.17 ± 0.05	1.13 ± 0.05	1.13 ± 0.02	1.14 ± 0.05	1.12 ± 0.05	1.12 ± 0.04
True Protein (g/dL)	0.96 ± 0.04	0.92 ± 0.03	0.93 ± 0.03	0.90 ± 0.03	0.90 ± 0.03	0.90 ± 0.03	0.91 ± 0.04	0.90 ± 0.04
Carbohydrate (g/dL)	8.51 ± 0.06	8.61 ± 0.09	8.46 ± 0.07	8.55 ± 0.12	8.47 ± 0.05	8.55 ± 0.10	8.41 ± 0.06	8.50 ± 0.12
Solids (g/dL)	11.3 ± 0.22	12.0 ± 0.35	12.4 ± 0.4	13.0 ± 0.4	12.7 ± 0.4	13.6 ± 0.5	15.6 ± 0.6	17.1 ± 0.8

Values are mean ± SEM. No significant differences were seen in milk protein, carbohydrate and solid content between OW/OB and Normal BMI women, and between the first, middle and last milk samples.

**Table 3:** Infant Characteristics (male and female combined).

	Normal (BMI 18.9–24.9) *n* = 9	OW/OB (BMI ≥ 25) *n* = 13	*p*-Value OW/OB vs. Normal
Male	6	4	
Female	3	7	
Birth Weight (g)	3235 ± 145	3413 ± 95	*p* = 0.0677
Birth Height (cm)	50.7 ± 0.7	50.2 ± 0.6	NS
Ponderal Index ^ (Birth)	2.4 ± 0.1	2.7 ± 0.1	*p* = 0.0573
Body Weight at Visit (g)	5032 ± 48	5058 ± 38	NS
Length at Visit (cm)	56.7 ± 0.7	56.5 ± 0.5	NS
Milk Intake per Feed (mL)	64.5 ± 6.4	90.0 ± 13.6	*p* < 0.05
Milk Intake (mL/kg body weight)	12.3 ± 1.2	17.6 ± 2.6	*p* < 0.05

Values are mean ± SEM. ^ Ponderal Index: 100 × weight (grams)/height^3^ (cm). NS = not significant.

## Data Availability

The data presented in this study are available from the corresponding author upon reasonable request. The data are not publicly available due to privacy or ethical restrictions.

## References

[1] Fryar C.D., Carroll M.D., Ogden C.L. (2021). Prevalence of Overweight, Obesity, and Severe Obesity among Adults Aged 20 and Over: United States, 1960–1962 through 2017–2018. Health E-Stats.

[2] Ho F.K., Celis-Morales C., Petermann-Rocha F., Parra-Soto S.L., Lewsey J., Mackay D., Pell J.P. (2021). Changes over 15 years in the contribution of adiposity and smoking to deaths in England and Scotland. BMC Public Health.

[3] Falkner B., Cossrow N.D.F.H. (2014). Prevalence of metabolic syndrome and obesity-associated hypertension in the racial ethnic minorities of the United States. Curr. Hypertens. Rep..

[4] Kit B.K., Kuklina E., Carroll M.D., Ostchega Y., Freedman D.S., Ogden C.L. (2015). Prevalence of and trends in dyslipidemia and blood pressure among US children and adolescents, 1999–2012. JAMA Pediatr..

[5] Nguyen D., Kit B., Carroll M. (2015). Abnormal Cholesterol among Children and Adolescents in the United States, 2011–2014. NCHS Data Brief.

[6] Mozaffarian D., Benjamin E.J., Go A.S., Arnett D.K., Blaha M.J., Cushman M., Das S.R., de Ferranti S., Despres J.P., Fullerton H.J. (2016). Heart Disease and Stroke Statistics—2016 Update: A Report from the American Heart Association. Circulation.

[7] Ismaeel A., Weems S., McClendon M., Morales F.E. (2018). Interventions Aimed at Decreasing Obesity in Hispanic Children in the First 1000 Days: A Systematic Review. J. Immigr. Minor. Health.

[8] Hales C.M., Fryar C.D., Carroll M.D., Freedman D.S., Ogden C.L. (2018). Trends in Obesity and Severe Obesity Prevalence in US Youth and Adults by Sex and Age, 2007–2008 to 2015–2016. JAMA.

[9] Hauerslev M., Narang T., Gray N., Samuels T.A., Bhutta Z.A. (2022). Childhood obesity on the rise during COVID-19: A request for global leaders to change the trajectory. Obesity.

[10] Guo S.S., Chumlea W.C. (1999). Tracking of body mass index in children in relation to overweight in adulthood. Am. J. Clin. Nutr..

[11] Lake J.K., Power C., Cole T.J. (1997). Child to adult body mass index in the 1958 British birth cohort: Associations with parental obesity. Arch. Dis. Child..

[12] Zhang T., Whelton P.K., Xi B., Krousel-Wood M., Bazzano L., He J., Chen W., Li S. (2019). Rate of change in body mass index at different ages during childhood and adult obesity risk. Pediatr. Obes..

[13] Dennison B.A., Edmunds L.S., Stratton H.H., Pruzek R.M. (2006). Rapid infant weight gain predicts childhood overweight. Obesity.

[14] Dubois L., Girard M. (2006). Early determinants of overweight at 4.5 years in a population-based longitudinal study. Int. J. Obes..

[15] Stettler N., Kumanyika S.K., Katz S.H., Zemel B.S., Stallings V.A. (2003). Rapid weight gain during infancy and obesity in young adulthood in a cohort of African Americans. Am. J. Clin. Nutr..

[16] Nader P.R., O’Brien M., Houts R., Bradley R., Belsky J., Crosnoe R., Friedman S., Mei Z., Susman E.J. (2006). Identifying risk for obesity in early childhood. Pediatrics.

[17] Ong K.K., Ahmed M.L., Emmett P.M., Preece M.A., Dunger D.B. (2000). Association between postnatal catch-up growth and obesity in childhood: Prospective cohort study. BMJ.

[18] Stettler N., Zemel B.S., Kumanyika S., Stallings V.A. (2002). Infant weight gain and childhood overweight status in a multicenter, cohort study. Pediatrics.

[19] Whitaker R.C. (2004). Predicting preschooler obesity at birth: The role of maternal obesity in early pregnancy. Pediatrics.

[20] Ehrenthal D.B., Maiden K., Rao A., West D.W., Gidding S.S., Bartoshesky L., Carterette B., Ross J., Strobino D. (2013). Independent relation of maternal prenatal factors to early childhood obesity in the offspring. Obstet. Gynecol..

[21] Sridhar S.B., Darbinian J., Ehrlich S.F., Markman M.A., Gunderson E.P., Ferrara A., Hedderson M.M. (2014). Maternal gestational weight gain and offspring risk for childhood overweight or obesity. Am. J. Obstet. Gynecol..

[22] Oken E., Taveras E.M., Kleinman K.P., Rich-Edwards J.W., Gillman M.W. (2007). Gestational weight gain and child adiposity at age 3 years. Am. J. Obstet. Gynecol..

[23] Diesel J.C., Eckhardt C.L., Day N.L., Brooks M.M., Arslanian S.A., Bodnar L.M. (2015). Is gestational weight gain associated with offspring obesity at 36 months?. Pediatr. Obes..

[24] Bider-Canfield Z., Martinez M.P., Wang X., Yu W., Bautista M.P., Brookey J., Page K.A., Buchanan T.A., Xiang A.H. (2017). Maternal obesity, gestational diabetes, breastfeeding and childhood overweight at age 2 years. Pediatr. Obes..

[25] Desai M., Ross M.G. (2020). Maternal-infant nutrition and development programming of offspring appetite and obesity. Nutr. Rev..

[26] Desai M., Ferrini M.G., Han G., Narwani K., Ross M.G. (2020). Maternal High Fat Diet Programs Male Mice Offspring Hyperphagia and Obesity: Mechanism of Increased Appetite Neurons via Altered Neurogenic Factors and Nutrient Sensor AMPK. Nutrients.

[27] Anstey E.H., Chen J., Elam-Evans L.D., Perrine C.G. (2017). Racial and Geographic Differences in Breastfeeding—United States, 2011–2015. MMWR Morb. Mortal. Wkly. Rep..

[28] Beauregard J.L., Hamner H.C., Chen J., Avila-Rodriguez W., Elam-Evans L.D., Perrine C.G. (2019). Racial Disparities in Breastfeeding Initiation and Duration among U.S. Infants Born in 2015. MMWR Morb. Mortal. Wkly. Rep..

[29] Centers for Disease Control and Prevention (2013). Progress in increasing breastfeeding and reducing racial/ethnic differences—United States, 2000–2008 births. MMWR Morb. Mortal. Wkly. Rep..

[30] Lapillonne A., Bronsky J., Campoy C., Embleton N., Fewtrell M., Fidler Mis N., Gerasimidis K., Hojsak I., Hulst J., Indrio F. (2019). Feeding the Late and Moderately Preterm Infant: A Position Paper of the European Society for Paediatric Gastroenterology, Hepatology and Nutrition Committee on Nutrition. J. Pediatr. Gastroenterol. Nutr..

[31] Haschke F., Haiden N., Detzel P., Yarnoff B., Allaire B., Haschke-Becher E. (2013). Feeding patterns during the first 2 years and health outcome. Ann. Nutr. Metab..

[32] Duncan B., Ey J., Holberg C.J., Wright A.L., Martinez F.D., Taussig L.M. (1993). Exclusive breast-feeding for at least 4 months protects against otitis media. Pediatrics.

[33] Wright A.L., Holberg C.J., Martinez F.D., Morgan W.J., Taussig L.M. (1989). Breast feeding and lower respiratory tract illness in the first year of life. Group Health Medical Associates. BMJ.

[34] Hopkins D., Steer C.D., Northstone K., Emmett P.M. (2015). Effects on childhood body habitus of feeding large volumes of cow or formula milk compared with breastfeeding in the latter part of infancy. Am. J. Clin. Nutr..

[35] Huang J., Zhang Z., Wu Y., Wang Y., Wang J., Zhou L., Ni Z., Hao L., Yang N., Yang X. (2018). Early feeding of larger volumes of formula milk is associated with greater body weight or overweight in later infancy. Nutr. J..

[36] Leghi G.E., Netting M.J., Middleton P.F., Wlodek M.E., Geddes D.T., Muhlhausler A.B.S. (2020). The impact of maternal obesity on human milk macronutrient composition: A systematic review and meta-analysis. Nutrients.

[37] Ahuja J.K.C., Casavale K.O., Li Y., Hopperton K.E., Chakrabarti S., Hines E.P., Brooks S.P.J., Bondy G.S., MacFarlane A.J., Weiler H.A. (2022). Perspective: Human Milk Composition and Related Data for National Health and Nutrition Monitoring and Related Research. Adv. Nutr..

[38] Calvo-Lerma J., Selma-Royo M., Hervas D., Yang B., Intonen L., González S., Martínez-Costa C., Linderborg K.M., Collado M.C. (2022). Breast Milk Lipidome Is Associated with Maternal Diet and Infants’ Growth. Front. Nutr..

[39] Ding Y., Yang Y., Xu F., Ye M., Hu P., Jiang W., Li F., Fu Y., Xie Z., Zhu Y. (2021). Association between dietary fatty acid patterns based on principal component analysis and fatty acid compositions of serum and breast milk in lactating mothers in Nanjing, China. Food Funct..

[40] Ross M.G., Kobayashi K., Han G., Desai M. (2022). Modulation of Milk and Lipid Synthesis and Secretion in a 3-Dimensional Mouse Mammary Epithelial Cell Culture Model: Effects of Palmitate and Orlistat. Nutrients.

[41] Zaniqueli D., Oliosa P.R., Neves F.S., Pani V.O., Martins C.R., de Souza Peçanha M.A., Barbosa M.C.R., de Faria E.R., de Oliveira Alvim R., Mill J.G. (2019). Ponderal index classifies obesity in children and adolescents more accurately than body mass index z-scores. Pediatr. Res..

[42] Groh-Wargo S., Valentic J., Khaira S., Super D.M., Collin M. (2016). Human Milk Analysis Using Mid-Infrared Spectroscopy. Nutr. Clin. Pract..

[43] Hsieh W.Y., Williams K.J., Su B., Bensinger S.J. (2021). Profiling of mouse macrophage lipidome using direct infusion shotgun mass spectrometry. STAR Protoc..

[44] Su B., Bettcher L.F., Hsieh W.Y., Hornburg D., Pearson M.J., Blomberg N., Giera M., Snyder M.P., Raftery D., Bensinger S.J. (2021). A DMS Shotgun Lipidomics Workflow Application to Facilitate High-Throughput, Comprehensive Lipidomics. J. Am. Soc. Mass. Spectrom..

[45] Jenness R. (1979). The composition of human milk. Semin. Perinatol..

[46] Jensen R.G., Ferris A.M., Lammi-Keefe C.J., Henderson R.A. (1990). Lipids of bovine and human milks: A comparison. J. Dairy Sci..

[47] Bzikowska A., Czerwonogrodzka-Senczyna A., Weker H., Wesołowska A. (2018). Correlation between human milk composition and maternal nutritional status. Rocz. Panstw. Zakl. Hig..

[48] Brown K.H., Akhtar N.A., Robertson A.D., Ahmed M.G. (1986). Lactational capacity of marginally nourished mothers: Relationships between maternal nutritional status and quantity and proximate composition of milk. Pediatrics.

[49] Han S.M., Derraik J.G.B., Vickers M.H., Devaraj S., Huang F., Pang W.W., Godfrey K.M., Chan S.Y., Thakkar S.K., Cutfield W.S. (2023). A nutritional supplement taken during preconception and pregnancy influences human milk macronutrients in women with overweight/obesity and gestational diabetes mellitus. Front. Nutr..

[50] Emery W.B., Canolty N.L., Aitchison J.M., Dunkley W.L. (1978). Influence of sampling on fatty acid composition of human milk. Am. J. Clin. Nutr..

[51] Mizuno K., Nishida Y., Taki M., Murase M., Mukai Y., Itabashi K., Debari K., Iiyama A. (2009). Is increased fat content of hindmilk due to the size or the number of milk fat globules?. Int. Breastfeed. J..

[52] Takumi H., Kato K., Nakanishi H., Tamura M., Ohto N.T., Nagao S., Hirose J. (2022). Comprehensive Analysis of Lipid Composition in Human Foremilk and Hindmilk. J. Oleo Sci..

[53] De Paula M.V.Q., Grant M., Lanigan J., Singhal A. (2023). Does human milk composition predict later risk of obesity? A systematic review. BMC Nutr..

[54] Ross M.G., Desai M. (2014). Developmental programming of appetite/satiety. Ann. Nutr. Metab..

[55] Peto R., Whitlock G., Jha P. (2010). Effects of obesity and smoking on U.S. life expectancy. N. Engl. J. Med..

[56] Xi B., Mi J., Duan J.L., Yan S.J., Cheng H., Hou D.Q., Zhao X.Y. (2009). Familial clustering of obesity and the role of lifestyle factors among children in Beijing. Zhonghua Yu Fang. Yi Xue Za Zhi.

[57] Burke V., Beilin L.J., Dunbar D. (2001). Family lifestyle and parental body mass index as predictors of body mass index in Australian children: A longitudinal study. Int. J. Obes. Relat. Metab. Disord..

[58] Maksvytis A., Stakisaitis D. (2004). Impact of obesity on lipid profiles in middle-aged women. Medicina.

[59] Bays H.E., Toth P.P., Kris-Etherton P.M., Abate N., Aronne L.J., Brown W.V., Gonzalez-Campoy J.M., Jones S.R., Kumar R., La Forge R. (2013). Obesity, adiposity, and dyslipidemia: A consensus statement from the National Lipid Association. J. Clin. Lipidol..

[60] Franssen R., Monajemi H., Stroes E.S., Kastelein J.J. (2011). Obesity and dyslipidemia. Med. Clin. N. Am..

[61] Zhu Y., Zhu H., Dang Q., Yang Q., Huang D., Zhang Y., Cai X., Yu H. (2021). Changes in serum TG levels during pregnancy and their association with postpartum hypertriglyceridemia: A population-based prospective cohort study. Lipids Health Dis..

[62] Kallio M.J., Siimes M.A., Perheentupa J., Salmenperä L., Miettinen T.A. (1992). Serum cholesterol and lipoprotein concentrations in mothers during and after prolonged exclusive lactation. Metabolism.

[63] Mohammad M.A., Sunehag A.L., Haymond M.W. (2014). De novo synthesis of milk triglycerides in humans. Am. J. Physiol. Endocrinol. Metab..

[64] Mohammad M.A., Sunehag A.L., Haymond M.W. (2009). Effect of dietary macronutrient composition under moderate hypocaloric intake on maternal adaptation during lactation. Am. J. Clin. Nutr..

[65] Neville M.C., Picciano M.F. (1997). Regulation of milk lipid secretion and composition. Annu. Rev. Nutr..

[66] Smith S. (1980). Mechanism of chain length determination in biosynthesis of milk fatty acids. J. Dairy Sci..

[67] Donovan S., Dewey K., Novotny R., Stang J., Taveras E., Kleinman R., Raghavan R., Nevins J., Scinto-Madonich S., Kim J.H. (2020). Dietary Patterns During Lactation and Human Milk Composition and Quantity: A NESR Systematic Review. Curr. Dev. Nutr..

[68] Ward E., Yang N., Muhlhausler B.S., Leghi G.E., Netting M.J., Elmes M.J., Langley-Evans S.C. (2021). Acute changes to breast milk composition following consumption of high-fat and high-sugar meals. Matern. Child. Nutr..

[69] Xi Q., Liu W., Zeng T., Chen X., Luo T., Deng Z. (2023). Effect of Different Dietary Patterns on Macronutrient Composition in Human Breast Milk: A Systematic Review and Meta-Analysis. Nutrients.

[70] Keikha M., Bahreynian M., Saleki M., Kelishadi R. (2017). Macro- and Micronutrients of Human Milk Composition: Are They Related to Maternal Diet? A Comprehensive Systematic Review. Breastfeed. Med..

[71] Keikha M., Shayan-Moghadam R., Bahreynian M., Kelishadi R. (2021). Nutritional supplements and mother’s milk composition: A systematic review of interventional studies. Int. Breastfeed. J..

[72] Kim S.Y., Yi D.Y. (2020). Components of human breast milk: From macronutrient to microbiome and microRNA. Clin. Exp. Pediatr..

[73] Burnol A.F., Loizeau M., Girard J. (1990). Insulin receptor activity and insulin sensitivity in mammary gland of lactating rats. Am. J. Physiol..

[74] Ramos-Roman M.A., Lapidot S.A., Phair R.D., Parks E.J. (2012). Insulin activation of plasma nonesterified fatty acid uptake in metabolic syndrome. Arterioscler. Thromb. Vasc. Biol..

[75] Ramos P., Martín-Hidalgo A., Herrera E. (1999). Insulin-induced up-regulation of lipoprotein lipase messenger ribonucleic acid and activity in mammary gland. Endocrinology.

[76] Makker K., Zhang M., Wang G., Hong X., Aziz K.B., Wang X. (2022). Maternal and fetal factors affecting cord plasma leptin and adiponectin levels and their ratio in preterm and term newborns: New insight on fetal origins of metabolic dysfunction. Precis. Nutr..

[77] Feuermann Y., Mabjeesh S.J., Shamay A. (2004). Leptin affects prolactin action on milk protein and fat synthesis in the bovine mammary gland. J. Dairy Sci..

[78] Neville M.C., McFadden T.B., Forsyth I. (2002). Hormonal regulation of mammary differentiation and milk secretion. J. Mammary Gland. Biol. Neoplasia.

[79] Yonezawa T., Yonekura S., Kobayashi Y., Hagino A., Katoh K., Obara Y. (2004). Effects of long-chain fatty acids on cytosolic triacylglycerol accumulation and lipid droplet formation in primary cultured bovine mammary epithelial cells. J. Dairy Sci..

[80] Butts C.A., Hedderley D.I., Herath T.D., Paturi G., Glyn-Jones S., Wiens F., Stahl B., Gopal P. (2018). Human Milk Composition and Dietary Intakes of Breastfeeding Women of Different Ethnicity from the Manawatu-Wanganui Region of New Zealand. Nutrients.

[81] Su L.L., Chelvi S.K.T., Lim S.L., Chen Y., Tan E.A., Pai N.N., Gong Y.H., Foo J., Rauff M., Chong Y.S. (2010). The influence of maternal ethnic group and diet on breast milk fatty acid composition. Ann. Acad. Med. Singap..

[82] Perez M.R., de Castro L.S., Chang Y.S., Sañudo A., Marcacine K.O., Amir L.H., Ross M.G., Coca K.P. (2021). Breastfeeding Practices and Problems among Obese Women Compared with Nonobese Women in a Brazilian Hospital. Women’s Health Rep..

[83] Gaillard R., Steegers E.A., Duijts L., Felix J.F., Hofman A., Franco O.H., Jaddoe V.W. (2014). Childhood cardiometabolic outcomes of maternal obesity during pregnancy: The Generation R Study. Hypertension.

[84] Meier P.P., Lysakowski T.Y., Engstrom J.L., Kavanaugh K.L., Mangurten H.H. (1990). The accuracy of test weighing for preterm infants. J. Pediatr. Gastroenterol. Nutr..

[85] Lemes S.F., de Souza A.C.P., Payolla T.B., Versutti M.D., de Fátima da Silva Ramalho A., Mendes-da-Silva C., Souza C.M., Milanski M., Torsoni A.S., Torsoni M.A. (2018). Maternal Consumption of High-fat Diet in Mice Alters Hypothalamic Notch Pathway, NPY Cell Population and Food Intake in Offspring. Neuroscience.

[86] Larnkjær A., Ong K.K., Carlsen E.M., Ejlerskov K.T., Mølgaard C., Michaelsen K.F. (2018). The Influence of Maternal Obesity and Breastfeeding on Infant Appetite- and Growth-Related Hormone Concentrations: The SKOT Cohort Studies. Horm. Res. Paediatr..

[87] Norrish I., Sindi A., Sakalidis V.S., Lai C.T., McEachran J.L., Tint M.T., Perrella S.L., Nicol M.P., Gridneva Z., Geddes D.T. (2023). Relationships between the Intakes of Human Milk Components and Body Composition of Breastfed Infants: A Systematic Review. Nutrients.

[88] Nikniaz L., Jr Mahdavi R., Arefhoesseini S.R., Sowti Khiabani M. (2009). Association between fat content of breast milk and maternal nutritional status and infants’ weight in Tabriz, Iran. Malays. J. Nutr..

[89] Ruggiero C.F., Hohman E.E., Birch L.L., Paul I.M., Savage J.S. (2021). INSIGHT responsive parenting intervention effects on child appetite and maternal feeding practices through age 3 years. Appetite.

